# Mechanical Performance of Recycled Reinforced Polyamide from Rejected Railway Fastenings Flanged Plates

**DOI:** 10.3390/polym14224940

**Published:** 2022-11-15

**Authors:** Soraya Diego, Jose Casado, Isidro Carrascal, Jose Sainz-Aja, Diego Ferreño

**Affiliations:** LADICIM (Laboratory of Materials Science and Engineering), University of Cantabria, E.T.S. de Ingenieros de Caminos, Canales y Puertos, Av/Los Castros 44, 39005 Santander, Spain

**Keywords:** recycling, reinforced polyamide, railway fasteners, viscosity, fatigue

## Abstract

The superstructure of modern railway lines uses tons of technical polymeric material spread along the track with mechanical, insulating and damping functions. Many of these parts are rejected because they do not pass the quality controls, generating a large accumulation of plastic waste of high economic value. Therefore, this study is aimed at determining the optimum degree of recyclability by mechanical crushing of geometrically defective (and so rejected) railway fastenings flanged plates injected with short fiberglass-reinforced polyamide. After recycling, the material must guarantee its physical and mechanical properties required to ensure the future in-service conditions of the highly responsible components that guarantee the maintenance of the railway gauge. Viscosity, mechanical properties (tensile test), Charpy and fracture toughness as well as fatigue performance were determined for ten successive recyclings. It has been found that the drop of viscosity is the most restrictive limitation, allowing three recyclings of the material. All the properties measured have experienced a noticeable reduction after 10 recyclings. Specifically, viscosity is reduced by 15%, ultimate strength by 70%, yield stress by 41% strain under maximum load lost by 70%, Young’s modulus lost by 38%, Charpy impact strength by 70%, fatigue resistance by 69% and fracture toughness lost by 80%. With the development of this study and taking into account that the market price of the flanged plates is valued at approximately 8 k€/km, of which around 5 k€/km corresponds to the raw material, the recovery of this material not only represents a great environmental benefit but also an economic one.

## 1. Introduction

Global polymer production has steadily grown for the last 50 years, increasing from 1.7 million tonnes in 1950 to 360 million tonnes in 2018. The leading end-use markets for polymers are, respectively, packaging (39.9% of the total demand), construction (19.8%), automotive (9.9%) and electrical and electronic equipment (6.2%) [1]. Overall, 80% of the demand corresponds to six large families of resin types: polyethylene, polypropylene, polyvinyl chloride, polystyrene, polyethylene terephthalate and polyurethane. Polymeric materials have been integrated and have replaced other traditional materials in many highly technical industrial applications. These polymers have been generically referred to as engineering plastics and have proven their capability of meeting the highest technical requirements of various engineering sectors.

Railway transportation (passengers and freights) is one of the sectors where intensive investment in this type of technical material occurs. The performance of rail transport has increased significantly in recent decades due to the gradual introduction of high-speed rails (HSR) worldwide. Even though there is no single standard, new lines in excess of 250 km/h and existing lines exceeding 200 km/h are widely considered high speed. In 1964, the first HSR of the world was inaugurated connecting Tokyo and Osaka (515 km); nowadays, HSR is currently in operation in more than 20 countries, the high-speed network covering more than 35,000 km (with more than 25,000 additional km under construction).

The increase in the speed of rail transport has changed the concept of long distance due to the reduction in the duration of the trip. Hence, HSR is considered best suited for journeys of 150–900 km for which the train can beat air and car trip time. In contrast, this rise of speed supposes an important increase in the loads that the superstructure of the track must resist, due to which the design of its elements and the selection of materials acquires an enormous relevance.

The fastening system between the rail and the sleeper is the element of greatest responsibility in order to guarantee the comfort, safety and reliability that the railway must provide. HSR tracks incorporate several engineering polymeric elements in its fastening system. The main function of these reinforced polymeric fasteners is to reduce the dynamic forces that the rail transmits to the concrete sleeper and to minimize the corresponding vibrations. The reinforcement of polymeric materials has been shown to improve their mechanical performance as demonstrated in studies by other authors [2,3,4,5]. Moreover, this system helps electrically isolating the rails as they conduct electrical signals to control the traffic of the line.

**Figure 1:**
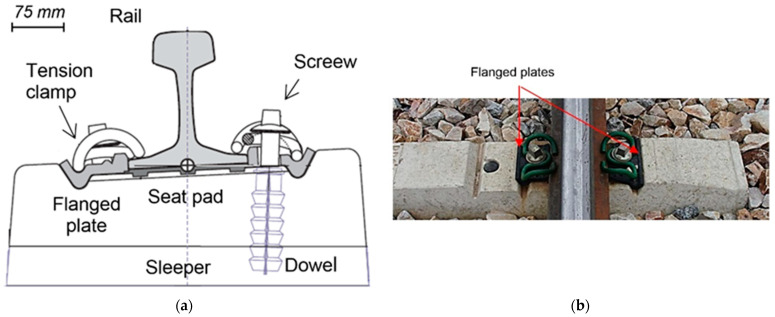
High-speed railway fastening system (VM). (**a**) Scheme of the fastening system. (**b**) Real picture of the fastening system.

In the Vossloh^TM^ Modified (VM) fastening system, widely used in HSR, the rail is fixed to the concrete sleeper by means of a couple of steel bolts screwed to a dowel inserted in the sleeper (see Figure 1a). Bolts are placed on both sides of the rail, so they can be external or internal depending on their position in relation to the axis of the track. As can be seen in Figure 1b, the VM fastening system includes two prestressed clips (that provide the track with tightness and elasticity, preventing the displacements or rotations of the rail), one seat pad that absorbs the vibrations generated by the passing of the trains, and two flanged plates that keep the track gauge and restraint track sideways.

Additional advantages derived from the use of elastomeric materials (rail seat pad) and high-performance technical polymers (flanged plate and dowel) in the fastening system rely on their dielectric nature as well as their excellent noise and vibration damping properties which guarantee the comfort of the passengers. Despite this, these polymers are not exempt from some inconveniences related to the cost of raw materials and the difficulties in the manufacturing process.

This research is focused on the properties of the flanged plates (see Figure 1b) which are manufactured by injection molding with polyamide 6.6, reinforced (35% weight) with short fiberglass (average length 200 micras). The final product must satisfy the requirements of the Technical Specification of the Administrator of Railway Infrastructures (TS-ADIF) [1]. Therefore, prior to be placed on the track, the flanged plates must be characterized to verify the physical and mechanical behavior. The parts used in the approval design and quality control tests as well as the residues that are generated during molding (such as risers) and off-spec materials are deposited in landfills, which has a significant environmental impact. This disposed material can reach around the 10% of the production. The hypothesis of this study is that these materials can be reused to fabricate new parts fulfilling all the required specifications, with beneficial environmental and economic impacts. Thus, the market price of the flanged plates for a track section amounts to ≈8k€/km of which ≈5 k€/km corresponds to the raw material.

The recycling process involves recovering the value of a material in the form of energy or reusable material. The recovery of plastic materials requires the meticulous cleaning and efficient sorting of each type of plastic. Once separated and sorted, plastic waste can be treated by four types of methods: mechanical recycling, chemical recycling, biological recycling and energy recovery. For this work, mechanical recycling was applied by grinding the plastic waste into small granules and then forming them. Mechanical recycling is a simple and economic process that requires crushing the material to be subsequently molded without any chemical additive. This procedure may be accompanied by the degradation of the quality of the material, which could reduce the lifespan of the component and/or its applicability under certain loading conditions [6,7,8]. For this reason, it is necessary to perform a physical and mechanical characterization to verify whether the materials injected with recycled material are suitable for the same industrial use or not.

The possible degradation derived from the mechanical recycling of polyamide 6.6 reinforced (35% weight) with short fiberglass motivates this study. The experimental scope is aimed at determining the evolution undergone by the physical and mechanical properties as a function of the number of recyclings the material was subjected to. The viscosity was selected as the most relevant physical parameter; regarding the mechanical properties, tensile, Charpy, fracture and fatigue tests were performed. Finally, a fractographic study was carried out by means of Scanning Electron Microscopy (SEM) as well as an analysis of the internal microstructure of the samples through micro-computed tomography (µCT).

## 2. Material and Methods

### 2.1. Material

The material used in the study comes from HSR flanged plates not previously placed on the track. The raw material (polyamide 6.6, reinforced (35% weight) with short fiberglass (average length 200 micras) was supplied by DuPont, BASF and DSM, respectively; see Table 1 and Table 2. Before molding, according to the recommendations of the specialist raw material manufacturer CEBUTOR PLASTICOS, the plates were cleaned and dried in a circulating air oven at 100 °C for 7 days (this is important, since polyamide is a hygroscopic material). After drying, the plates in the as-received condition were grinded in a mill with steel blades until a maximum particle size of 5–6 mm, which is similar to the raw material particle size. Then, the grinded material was injected (no stabilizing additives, antioxidants, bonding agents or other products that would help not to degrade the material or improve its properties or new raw material were added, i.e., only crushed material was injected) into standard tensile (type B) [9], Charpy impact [10] and fracture toughness [11] test specimens. The specimens were molded using an Arburg Allrounder 221 K injection molding machine with a clamping force of 35 tonnes, a maximum injection capacity of 49 cm^3^ and a screw diameter of 25 mm. The optimal injection parameters, which are summarized in Table 3, were determined following the guidelines established in the current standards [12,13], the injection recommendations published by the manufacturer of the raw material [14,15], the specifications of the injection machine and the injection variables published by other researchers [16,17,18].

The specimens in the as-received condition were referred to as R0 condition; the specimens obtained after injecting the grinded material were referred to as R1 condition. This same notation was applied after successive grindings and injections (up to a total of 10), giving rise to the experimental families R0–R10. All specimens (tensile, Charpy and fracture) were tested in the dry-as-molded (DAM) condition.

### 2.2. Viscosity

The mechanical properties of fiber-reinforced polyamide are strongly dependent on the viscosity of its matrix. The repetitive application of heating and shear stresses due to the injection during the molding process of the specimens leads to the irreversible breaking of the molecular chains of the matrix, which is reflected in a reduction in the viscosity of the material. For this reason, viscosity is a measure of the deterioration undergone by the material as a result of its recycling as well as an indicator of its ability to resist mechanical actions. The viscosity measurements were carried out according to the EN ISO 307 standard [19] to obtain the viscosity number (VN).

### 2.3. Mechanical Tests

To assess the influence of recycling on the mechanical behavior of the material, four experimental methods have been employed: tensile test, accelerated fatigue test, Charpy impact test and fracture toughness test, as they are considered to be the most representative tests affecting the mechanical and fracture behavior of the material according to the material performance in-service. The environmental conditions in all cases were a relative humidity of 50 ± 5% and a temperature of 23 ± 2 °C.

**Figure 2:**
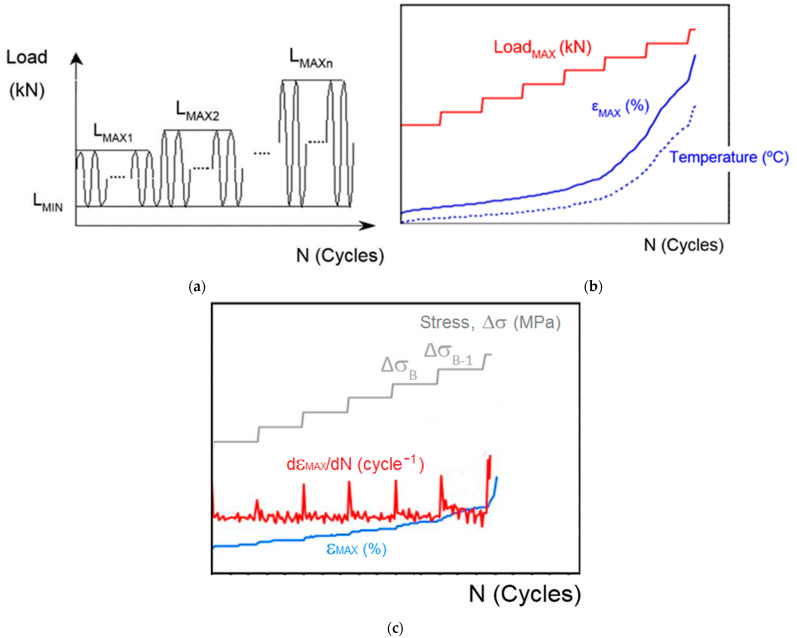
Schematic description of the accelerated fatigue LOCATI test. (**a**) Blocks of loading, (**b**) parameters measured and (**c**) definition of fatigue strength.

The static mechanical response was measured through tensile tests following the EN ISO 527-4 standard [9]. Three tests were performed for each of the material conditions (R1-R10). The accelerated fatigue LOCATI test [20,21] was employed to determine the fatigue limit; one of the main advantages of this method is that it allows the fatigue performance to be determined with one single test per material condition (therefore, a total of ten tests were conducted for families R1-R10). As can be seen in Figure 2a, the LOCATI test consists of the successive application of a series of blocks of oscillating loading, all of them with a constant number of cycles. The minimum load remains constant throughout the entire test; however, the maximum load increases progressively, from block to block. The load amplitude in the first block is very small, lower than the fatigue limit of the material. During the process, the maximum strain for the maximum load (ε_MAX_) of every cycle is recorded as well as the surface temperature of the specimen; see Figure 2b. As depicted in Figure 2c, a transient increase in the maximum strain occurs with each change of block, which is followed by the stabilization of this parameter. However, at a certain moment (stress amplitude Δσ_B-1_ in Figure 2c), this stabilization does not occur, which means that the fatigue limit of the material was exceeded. Conventionally, the fatigue strength of the material is identified as the stress amplitude in the immediately preceding block (Δσ_B_, Figure 2c) [22]. In this study, blocks of 20,000 sinusoidal wave cycles were applied at a frequency of 5 Hz, between initial tensile loads of 0.5 and 1.5 kN. The maximum load was increased by 0.25 kN per block. During the test, the strain values and surface temperature of the specimen were recorded (temperature was measured using infrared thermography). Both tensile and fatigue tests were conducted on an electric/hydraulic universal testing machine (SERVOSIS model ME-405/1 series 1709) with a load cell capacity of 5 kN. In order to determine the strain, an INSTRON extensometer with a gauge length of 25 mm was employed.

Impact tests were performed on notched Charpy impact specimens according to EN IS0 179 standard [10]. An instrumented Charpy pendulum with a load cell capacity of 5J (INSTRON IMPACTOR II type 7613.000 s/n 21581) was used.

Fracture toughness was obtained from three-point bending tests, following the ASTM D 5045 standard [11] with the same machine used for static and fatigue tests. The standard specimens used were SENB notched type with a thickness B = 4 mm, width W = 10 mm and an initial crack length, a, between 0.45 and 0.55 W; the specimens were placed on two cylindrical supports at a distance of 4 W. Two or three tests were conducted for each material condition (R1–R10) at a constant displacement rate of 10 mm/min.

### 2.4. Fractography

The fractographic analysis of the fracture surfaces of the specimens subjected to fatigue tests was performed by means of an optical microscope (Axio Imager microscope metallographic brand Z1M) as well as with an SEM (Zeiss EVO MA15) considering accelerating voltage (EHT:10 kV), Iprobe: 500 pA, aperture size: 100 µm and working depth (WD: 8 mm). A Balzers Union sputtering device, model SCD 040, was used to produce a conductive gold layer on the fracture sample surfaces.

### 2.5. Micro-Computed Tomography

The internal microstructure of the material was analyzed by using a Bruker Skyscan 1172 μCT equipped with a 80 kV and 100 μA X-ray source. The detail detectability was 2 μm measured through the image pixel size. After the scanning process, the reconstruction was performed employing the software NRecon-Bruker.

### 2.6. Analysis of Data and Statistics

Several material properties (viscosity, tensile parameters, Charpy resilience, fracture toughness and porosity) were plotted against the number of recyclings the specimens were subjected to. Linear regression (Y = β_0_ + β_1_·X) of the experimental results has been used to correlate the influence of recycling on these properties. The confidence bounds at the 95% significance level of the slopes of the fitting lines have been obtained to verify the statistical significance of the trends detected.

## 3. Results

The main results obtained in this study are summarized in Section 3.1, Section 3.2, Section 3.3, Section 3.4, Section 3.5, Section 3.6 and Section 3.7. Their analysis is developed in Section 4.

### 3.1. Viscosity

**Figure 3:**
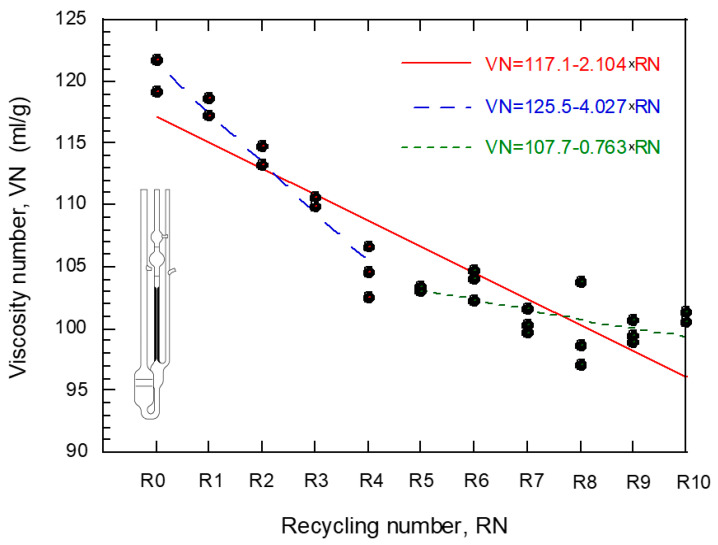
Evolution of the viscosity number, VN, with the number of recyclings, RN, applied.

The graph shown in Figure 3 represents the relationship between the number of recyclings, RN, and the viscosity number, VN. The data were fitted through a straight line (VN = β_0_ + β_1_·RN); the negative slope (β_1_ = −2.104) proves the detrimental effect of repetitive recyclings on the viscosity. Considering the distribution of points in the figure, a second fitting composed of two independent straight lines has been included: the first fitting includes points R0 to R4 (obtaining β_1_ = −4.027) and the second points R5 to R10 (with β_1_ = −0.763). The general impression is that this second fitting reproduces better the trend followed by the data. The variation undergone by the slope implies that the damaging effect on the viscosity derived from the recycling of the material attenuates from the fourth recycling. Table 4 gathers the coefficients, β_0_ and β_1_, of the linear fittings carried out, including their 95% confidence bounds as well as the value of R^2^. Notice that the confidence intervals of the slopes of groups R0–R4 and R5–R10 are disjoint, which proves the different behavior mentioned above.

### 3.2. Tensile Tests

**Figure 4:**
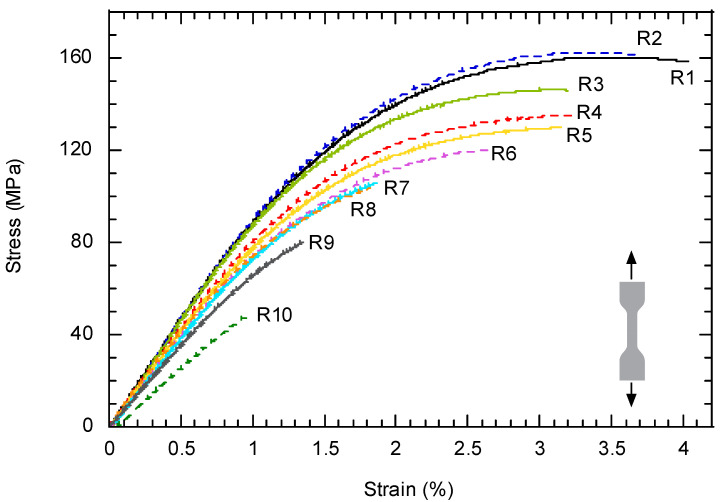
Experimental curves obtained in the tensile tests. Three tests were carried out for each material condition (number of recyclings); for clarity, only one of them was included in the graph.

**Figure 5:**
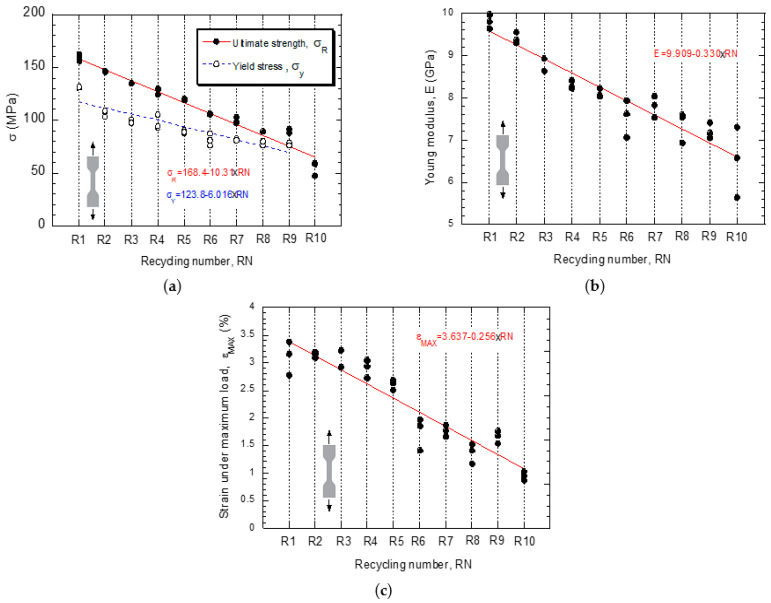
Change of mechanical properties as a function of the number of recyclings. (**a**) Yield stress, σ_Y_, and tensile strength, σ_R_, (**b**) Young’s modulus, E, evolution and (**c**) strain under maximum stress, ε_MAX_.

Figure 4 shows the experimental curves (stress vs. strain) obtained from the tensile tests on specimens fabricated after being subjected to different numbers of recyclings. Even though three tests were carried out for each material condition, for the sake of clarity, only one of them was included in the figure. The curves show the evident reduction undergone by three of the most relevant mechanical parameters, namely, the Young’s modulus, the tensile strength and the strain under maximum load, as the number of recyclings increases. In this sense, in Figure 5, the values of these three mechanical parameters are represented as a function of the number of recyclings. The general impression is that the process of damage undergone by the material continues progressively, without reaching a steady state after 10 recyclings. The coefficients, β_0_ and β_1_ of the linear fittings, including their 95% confidence bounds, as well as the value of R^2^ are summarized in Table 5.

### 3.3. Charpy Impact Tests

**Figure 6:**
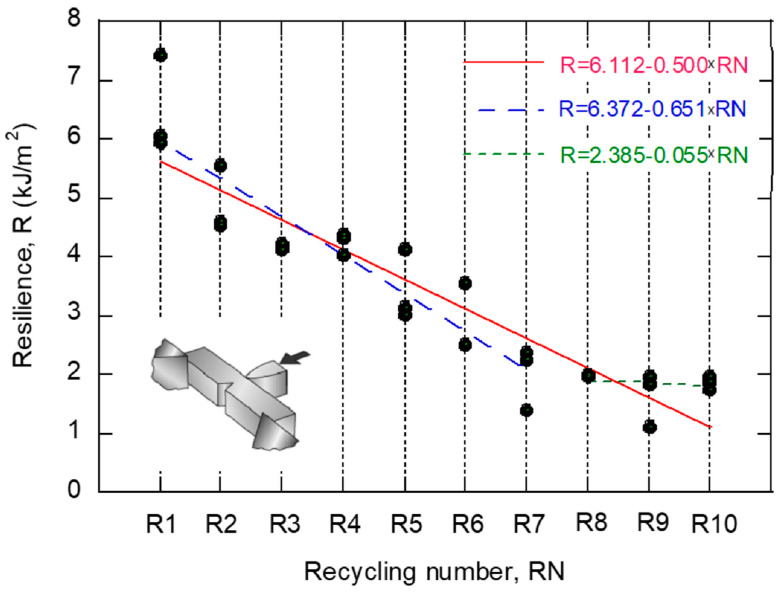
Evolution of impact strength with recycling number.

The Charpy test determines the amount of energy absorbed by a material during fracture. Figure 6 collects the values of the Charpy resilience (energy absorbed per unit fracture cross-section) as a function of the number of recyclings, RN. An evident reduction in resilience is appreciated with the number of recyclings. A linear fitting is included in the figure, considering all the data, R1–R10; in this case, a slope β_1_ = −0.5 is obtained. Moreover, a bilinear fitting consisting of two independent straight lines was considered: the first line includes the points belonging to R0–R7 and the second includes those in R8–R10. Apparently, this second approach describes the trends more accurately. As can be seen, the damaging process attenuates, since the slope for group R0-R7 is β_1_ = −0.6511, whereas for group R8–R10, it is β_1_ = −0.055. Table 6 gathers the coefficients, β_0_ and β_1_, of the linear fittings carried out, including their 95% confidence bounds, as well as the values of R^2^. Notice that the confidence intervals of the slopes of groups R0–R4 and R5–R10 are disjoint.

### 3.4. LOCATI Fatigue Test

**Figure 7:**
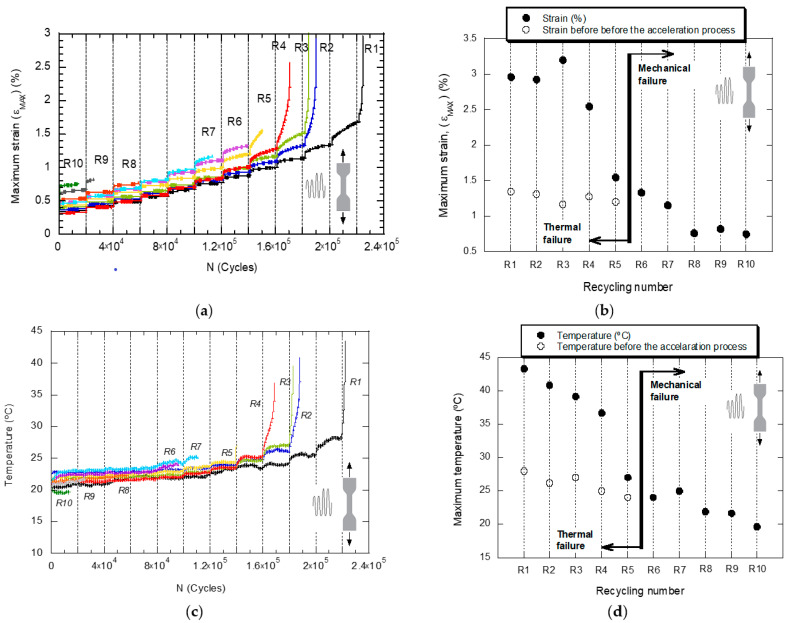
Evolution of the maximum strain, ε_MAX_, as a function of ((**a**) the number of fatigue cycles applied in the LOCATI test for the different material conditions (R1 to R10, (**b**) the recycling number) and evolution of the surface temperature of the sample as a function of ((**c**) the number of fatigue cycles applied in the LOCATI test for the different material conditions (R1 to R10) and (**d**) the recycling number).

**Figure 8:**
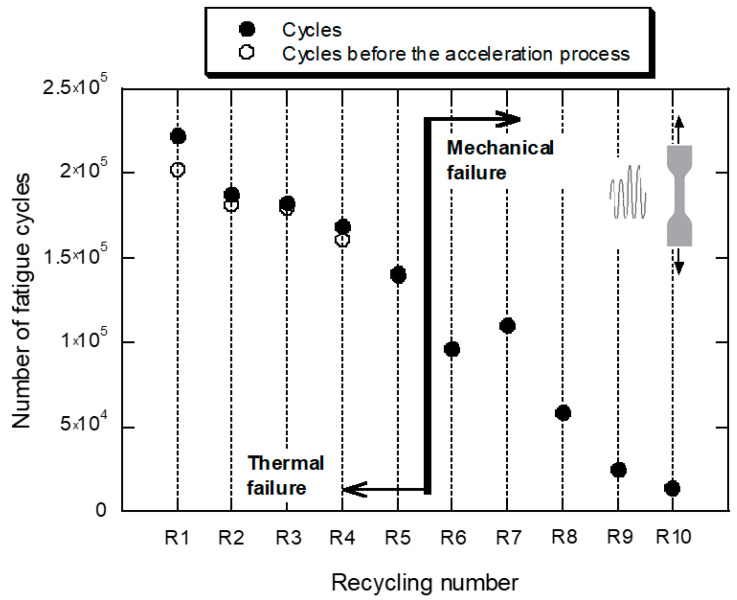
Evolution of the number of fatigue cycles as a function of the recycling number.

The graphs in Figure 7 show the evolution of the maximum strain, ε_MAX_, and the surface temperature of the specimen as a function of the number of fatigue cycles, for the ten recycling conditions (R1–R10). The graph in Figure 8 shows the number of fatigue cycles as a function of the recycling number. The trends contained in both figures point in the same direction: the ability of the material to resist fatigue loading is severely penalized by the application of successive recyclings.

### 3.5. Fracture Toughness Test

**Figure 9:**
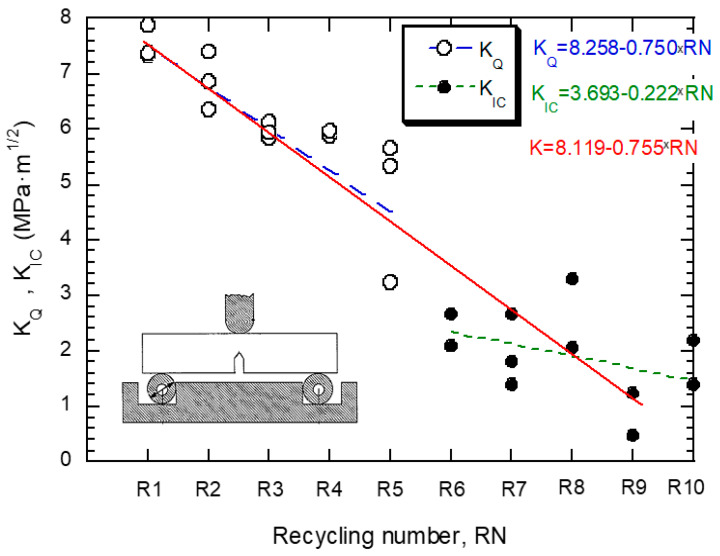
Evolution of fracture toughness with the number of recyclings.

The testing standard ASTM D5045-14 [11] establishes the procedure and conditions to be fulfilled to determine the plane–strain fracture toughness of plastic materials. In this sense, the thickness of the specimen, B, must exceed a minimum value to guarantee that fracture occurs under plane strain conditions, which is expressed in the following relation: B > 2.5·(K_IC_/σ_y_)^2^. The value of the stress intensity factor at fracture is referred to as K_Q_; only when the thickness condition is fulfilled (as well as other validity checks) may the K_Q_ value be quoted as a fracture toughness result, K_IC_. The thickness condition was not fulfilled for material conditions R1 to R5 but for the rest of them. Figure 9 shows the results of K_IC_ and K_Q_ for different numbers of applied recyclings. It can be observed that the toughness decreases as the number of recyclings increases, but this phenomenon mitigates from R6. For this reason, the data were fitted by means of a straight line as well as with two straight lines including families R1–R5 and R6–R10, respectively. The parameters derived from the fittings are collected in Table 7.

### 3.6. Fractographic Analysis

**Figure 10:**
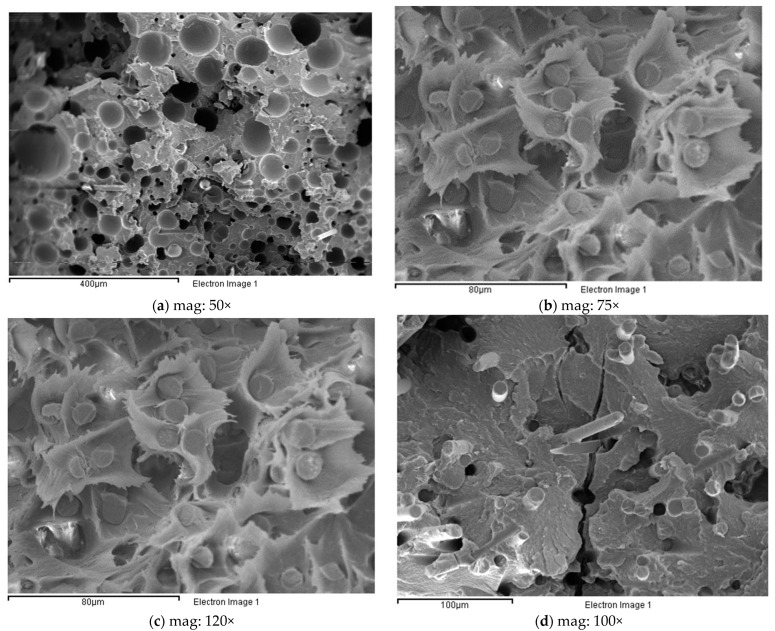
SEM fractographic analysis. (**a**) Fatigue failure surface of specimens R10. (**b**) Fatigue failure surface of specimens R9. (**c**) Low recycling number (R1) failure surface. (**d**) Crack growth mechanism for specimen R4.

The fracture surfaces of the samples were observed after the fatigue tests. The SEM micrographs in Figure 10a,b, show the fatigue failure surface of specimens R10 and R9, respectively; the large amount of porosity and the brittle appearance of the matrix surrounding the pores can be observed. In Figure 10b, the interconnection of pores through cracks, with a flat matrix surface, can be seen. These features are common to all the samples subjected to a large number of recyclings. In contrast, for the low recycling number shown in Figure 10c, which corresponds to R1, the large strain undergone by the matrix is the most remarkable property. Figure 10d shows the crack growth mechanism for specimen R4; in this case, a crack grows through the cavities left after the fibers are pulled off. This mechanism was present for families R1 to R4.

**Figure 11:**
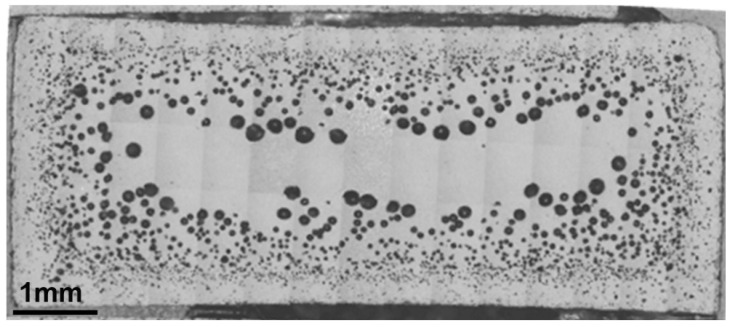
Porosity annular distribution in R10.

The existence of porosity was observed mainly in specimens subjected to a high number of recyclings. A specific study of the porosity distribution was carried out by cutting one fatigue tested specimen for each recycling condition into five different cross-sections. Then, they were analyzed with the optical microscope. It was found that the porosity is mainly manifested in high recycling conditions, over R4. The porosity increases with the distance from the point of injection; moreover, pores are arranged in an annular region as can be observed in Figure 11.

### 3.7. Micro-CT Analysis

**Figure 12:**
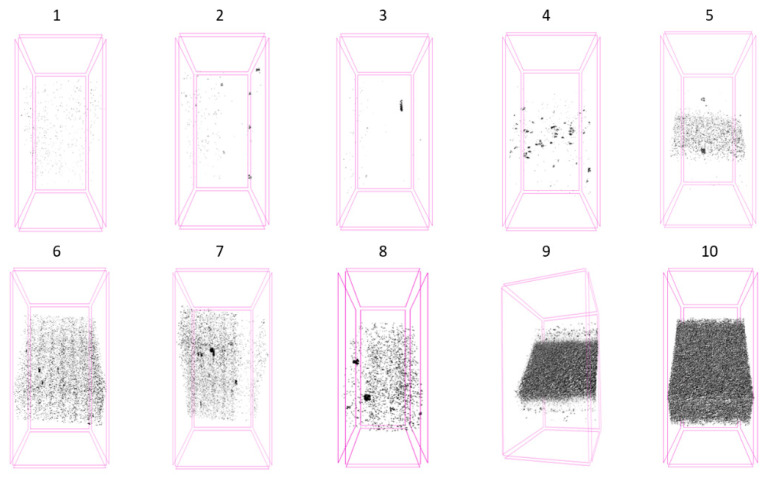
μCT pictures showing the distribution of porosity on samples as a function of the number of recyclings they were subjected to.

**Figure 13:**
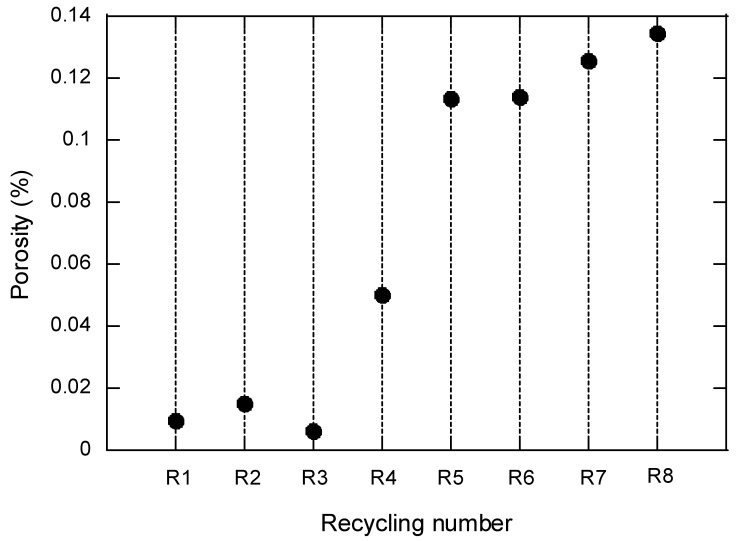
Porosity measured by means of μCT.

In order to define accurately the distribution of porosity in the fatigued samples, the ten recycling conditions were analyzed through μCT. Figure 12 shows the degree of porosity in a series of samples (32 mm^3^) extracted from the specimens. Notice that for R9 and R10, the level of porosity increases drastically. These results are represented in the graph of Figure 13, where different patterns can be distinguished: from R0 to R3, porosity is well below 0.02%; R4 corresponds to a transition region, giving way to the region between R5 and R8 where the porosity is in the range 0.10–0.14%; the results corresponding to R9 and R10 are not represented in the graph, since their porosity increases dramatically (around two orders of magnitude, 5.0 and 7.7%, respectively).

### 3.8. Fiber Length Analysis

**Figure 14:**
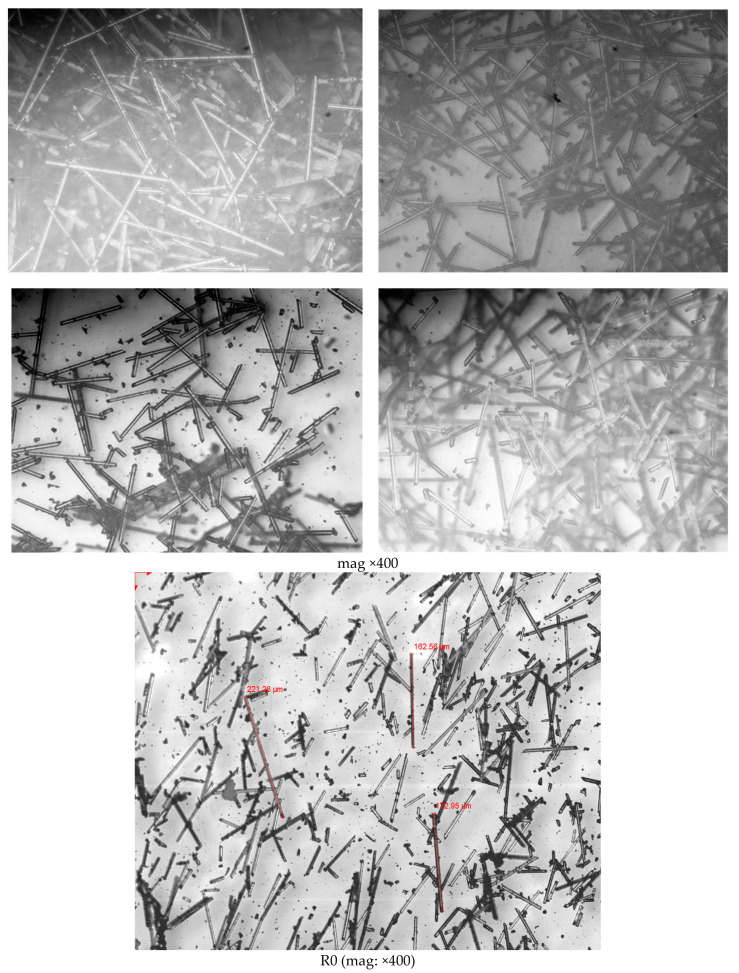
Optical microscope observation and measurement of fibers.

**Figure 15:**
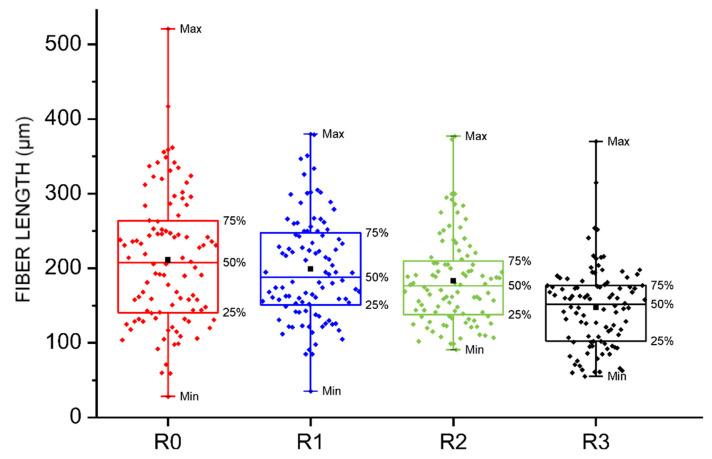
Short glass fiber length distribution.

To determine the length distribution of the glass fibers by successive recycling, the organic matter was volatilized by calcination according to ISO 1172 using a Carbolite Furnaces CSF1200 muffle, at 625 °C for 3 h. The process was carried out up to a third recycling as the degradation of the matrix, in terms of viscosity, was found to be excessive. The length of a population of 100 fibers/recycling was measured with the optical microscope. The photographs in Figure 14 show an example of the fibers from each recycling. The graph in Figure 15 shows the distribution of fiber lengths obtained in each case.

**Figure 16:**
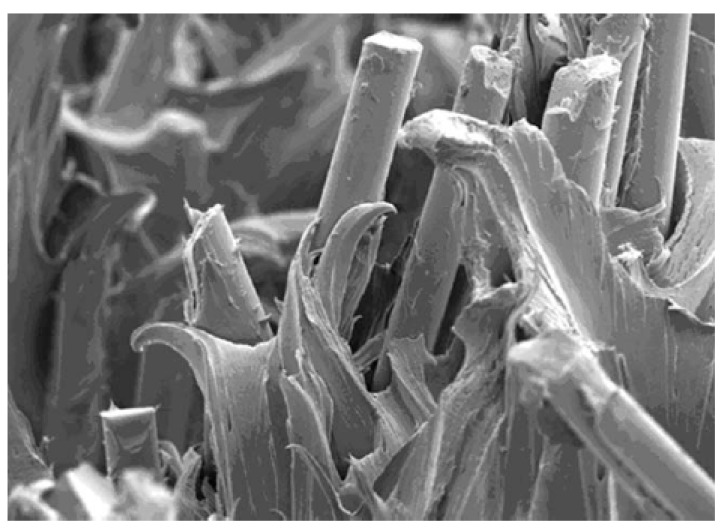
Internal structure of PA66GF35 (matrix interface fiber) X175 Mag.

The total fiber length decreases as it is recycled as a result of attrition due to successive grinding and re-injection. The breakage of a fiber into two or more shorter pieces causes an increase in the number of fiber ends, which act as stress concentration sites creating high shear stresses; in addition, these ends lack a bonding agent, increasing the fiber-matrix cohesion failure (see Figure 16).

## 4. Discussion

The experimental results obtained in this study reveal a noticeable deterioration of the physical and mechanical properties of polyamide reinforced with short glass fiber because of the application of successive recyclings based on mechanical grinding of the flanged plates. The decrease in viscosity due to a recycling process was observed by other authors [23,24,25]. From a microstructural viewpoint, this phenomenon is a consequence of the shortening of the polymeric chains caused by the successive injections. As shown in Figure 3, the reduction in viscosity is concentrated before the fifth recycling; after that, the microstructural damage is so pronounced that the effect of the subsequent recyclings is negligible. The viscosity of the polymer matrix is one of the factors that participates in the final behavior of the reinforced polyamide. For this reason, it is usual to impose conditions to guarantee an adequate mechanical behavior. For example, the technical specification published by ADIF establishes limits in this regard.

Even though the mechanical properties (tensile, Charpy, fatigue and fracture toughness) are all negatively affected by the process of recycling, the specific patterns are slightly different. The evolution of the mechanical properties represented in Figure 5 shows a process of continuous deterioration, not attenuated at any time. In this sense, it is worth noting that the mechanical response for this composite depends on the response of its constituents (matrix and fibers) as well as in the interaction between them. The viscosity number provides information about the deterioration undergone by the matrix but, as shown by Pedroso et al. [17], the mechanical behavior of the reinforce polyamide is strongly dependent on the length distribution of the fibers as well as on the specific conditions at the interface between fibers and matrix. One of the consequences of the mechanical grinding of the plates is the shortening of the glass fibers which, in turn, hampers the mechanical ability of the material. Fiber shortening has been studied and contrasted in several works including the authors of the present study [16,17,18,26,27]. In addition, other external factors play an important role, such as the porosity of the material [27]. As shown by means of micro CT (see Figure 13), the porosity of the material continuously increases with the number of recyclings (up to 8% in the case of R10 condition). The pores act as preferential paths for the propagation of the cracks. The appearance of a high number of pores is due to the incorporation of small particles of polyamide (powder size) produced in the grinding. These particles are thermally degraded by gasification during the injection process at high temperature. Therefore, sieving the crushed material before its injection would be advisable to eliminate, or at least, reduce its effect on the porosity.

The fracture surfaces analyzed by SEM show that there are regions of high deformation produced during the propagation of the crack and regions with brittle appearance due to the last and instantaneous breakage of the material in the last fatigue cycle. Moreover, the SEM pictures prove the existence of two different types of fracture micromechanisms after the LOCATI fatigue test: namely, the thermal failure and the mechanical failure. The SEM micrographs collected in Figure 10 show the two types of fracture. Thermal fatigue occurs from recyclings R1 to R4, while from R7 to R10, mechanical failure takes place. Recycling conditions R5 and R6 are the limit between these two types of failure. Figure 10a represents the mechanical fatigue failure, where the mechanism of crack growth in the brittle zone uses the pores as preferred paths (detail Figure 10b) and also, the temperature stabilizes in every loading block. In this case, the temperature never exceeds 25 °C, and the maximum strain is lower than 1% without any evidence of accelerated increase in the crack growth rate. Figure 10c, in contrast, represents the type of failure due to thermal fatigue, in which the cracks use the empty holes of the fibers that have lost their adherence with the matrix to advance along a plane perpendicular to the one of the break that finally prevails. In this case, the fracture occurs after a large number of cycles during which the levels of strain and temperature increase progressively. At this point, the surface temperature and the maximum strain reach critical values (≈40 °C and ≈3%, respectively) corresponding to the high crack growth rate that announces the imminence of the failure.

Currently, up to 10% of the raw material (polyamide 6.6 reinforced with short fiberglass) used to fabricate the flanged plates of the VM fastening system may go to waste for several reasons (surplus after molding, parts used for the assurance, etc.). This material represents up to 0.17 tons per kilometer of track. In this study, the degree of recyclability of this material has been studied in order to incorporate it back into the process of injection of flanged plates. The recycling process consisted in the mechanical crushing of the plates and the subsequent injection of the piece without adding any type of additive. The quality assurance of the recycled material obtained has been carried out through the study of the evolution of the physical and mechanical properties and the fractographic and tomographic analysis of the samples.

The physical-mechanical characterization carried out on standardized samples recycled up to 10 times shows a significant decrease in the mechanical properties of the composite material that alleviates after the fifth–sixth recycling. However, there are different regulations and specifications that limit possible modifications in the behavior of this material. For example, the technical specification promulgated by ADIF [28] states that the minimum viscosity must be greater than 90% of the viscosity of the virgin pellets. On the other hand, there are similar restrictions in the experimental results obtained in this research; it is admissible to subject the material up to three recyclings.

From the economic point of view of the manufacturer of the flanged plates, the recovery of this material would imply an estimated saving of 10% in costs of raw material. From the environmental point of view, recycling the plates by mechanical crushing is a clean process that reduces different types of pollutions such as air emissions of polymeric dust and volatile organic compounds (caprolactam and thermal oil), wastewater discharges with organic (cyclopentanone and hexamethylenediamine) and inorganic (mainly ammonia) content as well as solid waste. A detailed description of the sources of pollution related to the fabrication of polyamide can be found in [29].

Due to the recycling process, the glass fiber undergoes significant attrition and reduces its original average length (around 211 μm). The effect of up to three recyclings (R3) has been found to be a 30% reduction in the original average fiber length. In general, fiber breakage results in the formation of a greater number of shorter fiber ends which, in addition to being devoid of bonding agent, generate a greater number of discontinuities and potential defect nucleators in the recycled composite material.

**Figure 17:**
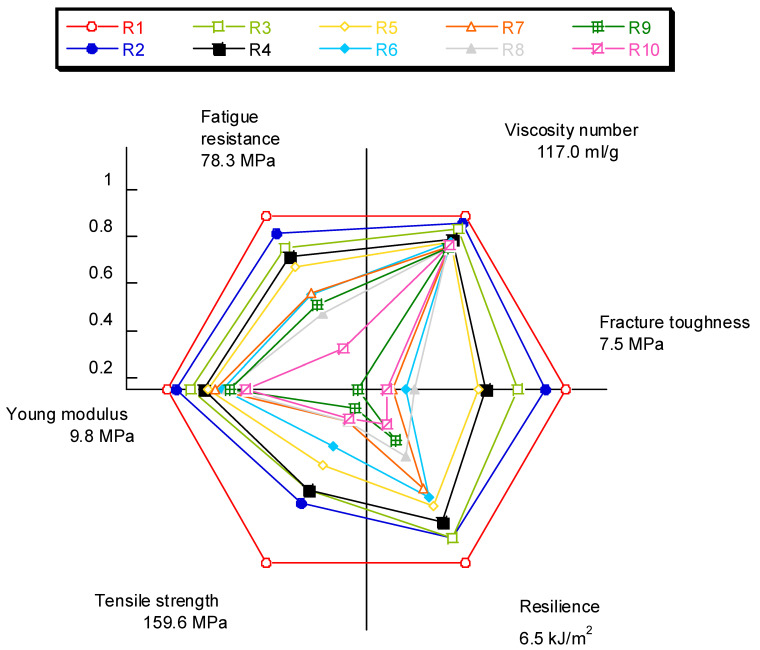
Radar graph showing the decrease in properties of the short glass fiber-reinforced polyamide as a function of the number of recyclings.

As a final summary, the radar graph in Figure 17 allows the evolution of the properties analyzed in this study to be appreciated as a function of the number of recyclings to which the material was subjected to.

## 5. Conclusions

- The decrease in viscosity is a consequence of the shortening of the polymeric chains caused by the successive injections and is concentrated before the fifth recycling. Having studied the effect on the properties of up to 10 recyclings and considering the restrictive values of the ADIF Technical Specification, up to a third recycling would be admissible.
- The mechanical properties are all detrimentally affected by the process of recycling. The mechanical behavior of the reinforce polyamide is strongly dependent on the shortening of the glass fibers and the length distribution of the fibers as well as on the specific conditions at the interface between the fibers and matrix. The effect of up to three recyclings (R3) has been found to be a 30% reduction in the original average fiber length.
- The porosity of the material, due to the incorporation of polyamide powder produced in the grinding, continuously increases with the number of recyclings acting as preferential paths for the propagation of the cracks.
- SEM analysis evidences the existence of two different types of fracture micromechanisms after the LOCATI fatigue test: namely, the thermal failure and the mechanical failure.
- From the economic point of view, the recovery of this material would imply an important saving of 37% in costs per kilometer.
- From the environmental point of view, recycling the plates by mechanical crushing reduces pollutions such as air emissions of polymeric dust and volatile organic compounds (caprolactam and thermal oil), wastewater discharges with organic (cyclopentanone and hexamethylenediamine) and inorganic (mainly ammonia) content as well as solid waste.

## Figures and Tables

**Table 1:** Properties of E-glass fiber (G. Lubin, Handbook of composites.: Van Nostrand Reinhold, 1983).

Chemical Composition	(Weight %)	Properties	Units	Value
SiO_2_	52.4	Tensile stress (*σ_f_)*	GPa	3.45
Al_2_O_3_. Fe_2_O_3_	14.4	Modulus of elasticity (*E_f_)*	GPa	72.50
CaO	17.2	Deformation at break (*ε_f_)*	%	3.30–4.48
MgO	4.6	Density (*ρ_f_*)	g/cm^3^	2.60
Na_2_O. K_2_O	0.8	*σ_f_/* *ρ* *_f_*	(GPa·cm^3^)/g	1.30
Ba_2_O_3_	10.6	*E_f_/* *ρ* *_f_*	(GPa·cm^3^)/g	28.00
		Fiber diameter (*d*)	μm	3–25
		Coeff. linear thermal expansion	10^−6^/K	5.00

**Table 2:** General properties of PA6.6GF35 (CAMPUS. (2013) CAMPUS plastics. (Online). http://www.campusplastics.com/ (accessed on 13 September 2022)).

Properties *DAM Condition*	Standard (ISO)	Units	DUPONT	BASF	DSM
Tensile strength	527-2	MPa	210	210	200
Young’s modulus	527-2	GPa	11.2	11.5	11
Elongation at break	527-2	%	3.2	3	3
Creep modulus 1 h	899-1	GPa	8.4	-	-
Resist. impact Charpy with notch (−30/23 °C)	179/1eA	kJ/m^2^	10/15	12/14	11/13
Rockwell hardness (M/R)	2039-2	-	105/125	-	-
Melting temperature	11357-3	°C	262	260	260
Glass transition temperature	11357-2	°C	80	-	-
Bending temperature (1.8 MPa)	75-1/2	°C	250	250	250
Specific heat	-	J/(kg·K)	2300	-	
Surface resistivity	IEC60093	Ω·m	>1.0 × 10^15^	-	-
Dissipation facto 1 MHz	IEC60250	–	0.014	0.020	0.014
Density	1183	g/cm^3^	1.41	1.41	1.41
Viscosity no.	307	cm^3^/g	130	145	155
Water absorption sat.	62	%	5.5	5	5.5

**Table 3:** Injection molding parameters employed to manufacture the specimens.

Parameter	Value
Mold temperature	Room temperature
Barrel temperature	300 °C
Melt temperature	300 °C
Cooling time	30 s
Total cycle time	50 s
Screw speed	29 m/min
Injection speed	18/25 (cm^3^/s)
Injection pressure	1800 bar
Back pressure	50/400/500 bar

**Table 4:** Summary of results obtained from the linear fitting (VN = β_0_ + β_1_·RN) of the viscosity (VN is expressed in mg/L). The coefficients β_0_ and β_1_ are accompanied with their 95% confidence bounds.

Data	β_0_	β_1_	R^2^
**R0–R10**	117.1 (114.7, 119.6)	−2.104 (−2.501, −1.708)	0.8269
**R0–R4**	125.5 (123.6, 127.3)	−4.027 (−4.741, −3.314)	0.9476
**R5–R10**	107 (102.1, 111.8)	−0.763 (−1.397, −0.129)	0.3224

**Table 5:** Summary of results obtained from the linear fitting (Y = β_0_ + β_1_·RN) of the experimental data. The coefficients β_0_ and β_1_ are accompanied with their 95% confidence bounds.

Property	β_0_	β_1_	R^2^
**E (MPa)**	9.909 (9.594, 10.22)	−0.3301 (−0.3801, −0.28)	0.8715
**σ_Y_ (MPa)**	123.8 (117.2, 130.3)	−6.016 (−7.191, −4.841)	0.8299
**σ_R_ (MPa)**	168.4 (161.4, 175.4)	−10.31 (−11.42, −9.192)	0.9302
**ε_MAX_**	3.637 (3.397, 3.877)	−0.2561 (−0.2942 −0.2179)	0.8755

**Table 6:** Summary of results obtained from the linear fitting (R = β_0_ + β_1_·RN) of the resilience. The coefficients β_0_ and β_1_ are accompanied with their 95% confidence bounds.

Group	β_0_	β_1_	R^2^
**R0–R10**	6.112 (5.613, 6.611)	−0.5000 (−0.5803, −0.4204)	0.8529
**R0–R7**	6.372 (6.032, 7.212)	−0.6511 (−0.7828, −0.5193)	0.8492
**R8–R10**	2.385 (−0.2234, 4.883)	−0.055 (−0.3375, 0.2275)	0.02938

**Table 7:** Summary of results obtained from the linear fitting (Y = β_0_ + β_1_·X) of the fracture toughness, K_Q_ or K_Ic_. The coefficients β_0_ and β_1_·are accompanied with their 95% confidence bounds.

Group	β_0_	β_1_	R^2^
**R1–R10**	8.119 (7.308, 8.93)	−0.7551 (−0.895, −0.6152)	0.8380
**R1–R5**	8.258 (7.088, 9.428)	−0.7504 (−1.103, −0.3977)	0.619
**R6–R10**	3.693 (0.6452, 6.741)	−0.222 (−0.6016, 0.1577)	0.1628

## Data Availability

Data will be made available upon request from the corresponding author.
